# Supplementary material for: Are carbon emissions trading and green financial instruments synergistic? -Comprehensive quantitative research based on content analysis
Source: PLoS One. 2024 Mar 7;19(3):e0298601. doi: 10.1371/journal.pone.0298601 (PMC10919601; doi:10.1371/journal.pone.0298601)
Supplement: S1 Data — (ZIP) [file pone.0298601.s001.zip › Data/地级市Z_F工作报告.zip!/┴╔─■/┴╔╤⌠╩╨/└┤╫╘╣1⁄2╓┌║┼í╛┬φ┐╦╩2╛▌═°í┐_2018.docx]

各位代表：

我代表市政府向大会报告工作，请予审议，并请市政协委员和列席会议的同志提出意见。

一、过去五年工作简要回顾和2017年主要工作

过去的五年，是辽阳发展进程中极不平凡的五年。五年来，市政府在省委、省政府和市委的正确领导下，团结带领全市人民，深入学习贯彻党的十九大精神，全面落实新发展理念和“四个着力”“三个推进”，坚持稳中求进工作总基调，主动适应把握引领经济发展新常态，紧紧围绕“四个辽阳”建设目标，统筹做好“稳增长、促改革、调结构、惠民生、防风险”各项工作，全市经济社会保持平稳健康发展。

转型升级迈出新步伐。供给侧结构性改革扎实推进，“三去一降一补”五大任务全面落实，三次产业结构为9.6：45.9：44.5。以芳烃及精细化工、铝合金精深加工两大千亿元产业基地和装备制造及汽车零部件产业集群为主导的工业发展格局基本形成。产学研合作提质升级，科技创新步伐加快。6个服务业集聚区全部跻身省级行列，冰雪温泉游和皮装裘皮购物游成为国家级特色品牌。国家现代农业示范区建设成效明显，在全省率先启动特色小镇建设，建成一批美丽乡村示范村。

改革开放实现新突破。持续推进“放管服”改革，取消、下放行政职权1300余项，相对集中行政审批权改革“辽阳模式”在全省推广，事中事后监管制度不断完善，政务服务便利化、规范化、标准化水平显著提高。营商环境持续改善，民营经济发展改革示范市建设深入推进。国资国企改革取得实效，妥善解决一批历史遗留问题。农村、社保、商事、财税、医药卫生、不动产登记等领域改革取得积极进展。外贸出口和引资规模持续扩大，开展国际经贸合作的国家达到106个。

生态宜居城市建设取得新成效。环境保护治理力度前所未有，主要污染物排放总量完成阶段性减排目标，辽阳的天更蓝、水更清、山更绿。推进新老城区融合发展，“跨河而立”的滨水型生态宜居城市初具形象。河东新城一期基本建成，道路、桥梁等基础设施日益完善，教育、医疗、文化等公共服务功能不断加强，太子河历史文化风光带成为城市靓丽风景线。老城区改建取得历史性突破，大规模棚改全面提升城市面貌，路网建设拉开城市骨架，历史文化景观建设厚植城市底蕴。坚持“创城促振兴、创建惠民生”，全力创建全国文明城市，市民整体素质和城市文明程度显著提升。

民生和社会事业得到新改善。五年累计投入67.1亿元，集中实施99项重点民生实事。教育教学质量稳步提升，5个城区全部晋升为国家义务教育发展均衡区。覆盖城乡的医疗卫生服务体系建设加快推进，人均卫生资源占有量居全省前列。文化体育惠民工程深入实施，市图书馆、辽阳大剧院、健身大厦等文体设施相继投入使用。脱贫攻坚扎实推进，就业再就业工作持续稳居全省前列，城乡社会保障体系日趋完善。城乡常住居民人均可支配收入年均分别增长8.1%和8.5%。

和谐社会建设开创新局面。平安辽阳建设深入推进，社会治安群众满意度逐年提高。信访总量、生产安全事故起数和死亡人数逐年下降，食品药品分级分类监管扎实推进，社会信用体系示范试点工作不断深入，社区网格化服务管理体系日趋完善，连续第八次荣获全国双拥模范城称号。

各位代表！刚刚过去的2017年，面对复杂变化的经济形势，我们迎难而上，砥砺奋进，各项工作取得积极成效，呈现经济筑底企稳、社会和谐稳定的良好局面，较好地完成了市人代会确定的目标任务。预计地区生产总值增长6.5%，一般公共预算收入增长14%，固定资产投资增长3%，城乡常住居民人均可支配收入分别增长6.7%和6.5%。

一年来，我们重点抓了以下八项工作：

（一）全力推进重点项目建设，发展后劲持续增强

深入开展“项目年”活动。建立项目审批绿色通道，妥善解决要素瓶颈。实施124个投资亿元以上的结构调整、基础设施和民生工程项目，23个项目竣工投产。96个“四个辽阳”重点项目全面完成年度建设任务。

多渠道筹集建设资金。推动政府和社会资本合作，引进社会资本33亿元。扩大有效信贷投放，金融机构本外币各项贷款余额1367亿元，比年初增加186亿元。发挥融资平台作用，实现融资12.8亿元。加强政府与金融机构合作，签订融资协议160亿元。积极争取上级资金支持，全年到位资金83亿元。

大力开展招商引资。明确主攻方向，精准出台招商政策，强力助推项目落地。采取高层次、专业化、重点招商相结合的方式，开展“走出去、引进来”招商活动360余次。坚持与智者同列、与巨人同行、与战略投资者合作，同中核集团、中铁中基等大型企业签订400多亿元的投资协议，促成50个项目签约。全年引进国内资金110亿元，实际利用外资7500万美元。

（二）调整优化工业结构，新旧动能加速转换

倾力帮扶实体经济。深入落实“营改增”等结构性减税政策，为企业让税10.2亿元。清理整顿涉企收费项目，为企业减负1.4亿元。执行直供电政策，取消指标限额，放宽准入范围，为企业节约电费1亿元。盘活利用闲置厂房10万平方米。采取发放“助保贷”、解决“过桥”资金、股权质押登记等手段，多措并举帮助企业融资130亿元。深入实施企业培育工程，完成“个转企”500余家，新增规模以上企业30家，销售收入亿元以上企业达到70家。

加快优化产业结构。辽化大技改项目设备全部安装就位，药用辅料一期满负荷生产，高档分散染料项目加快推进，芳烃及精细化工产业丰厚度进一步提升。铝合金精深加工系列项目投产或部分投产，建筑模板、全铝轿车车身、特种车身、全铝家具等深加工项目产能逐步释放。航空密封件产品正式下线，新能源汽车电机及电驱动总成项目加快推进，汽车轻量化材料、LED汽车灯等项目相继签约，装备制造及汽车零部件产业集群不断发展壮大。钢铁产业转型升级项目效益显著提升。淘汰钢铁落后产能446万吨，化解煤炭产能5万吨、水泥产能134万吨，为产业发展腾出空间。产业园区建设取得积极进展，辽阳经济开发区在省综合考评中列第五位，灯塔、弓长岭经济开发区跻身省级行列。新增省级智能制造试点示范企业5家，建成自动化和数字化车间5个，促进“两化”融合发展。二号首长

着力推进科技创新。组建5个校企协同创新骨干联盟，市科技创新服务中心投入运行，促成科技成果转化30项。加强创新平台建设，“辽宁钢铁共性技术创新中心”正式揭牌，建成精细化工中试基地，组建国家、省重点实验室各1家，新增省级技术中心6个。全面启动科技金融工作，国家知识产权质押融资试点市建设深入推进。引进国家级研发团队2个，新增高新技术企业20家。

（三）促进服务业提档升级，支撑作用日益凸显

大力发展生产性服务业。成功引进3家外埠金融机构，金融业增加值增长12%。全力培育6个电商基地，入驻企业已达205家。农村电子商务加快发展，邮乐购店和益农信息社实现乡村全覆盖。城乡物流配送体系初具规模，2家企业荣获全省首批农产品冷链物流示范企业。农产品现代流通体系和肉菜流通追溯体系两个试点市建设加快推进，万隆公益性农产品批发市场交易中心竣工。

加快发展生活性服务业。东京城遗址公园初具形象，燕州城景区基础设施不断完善，太子河生态路全线贯通。弓长岭国家全域旅游示范区加速创建，佟二堡成功申报国家4A级购物旅游景区。深入实施“后备箱”工程，28项商品纳入“后备箱”名录，房车营地项目落地。全国养老服务业综合改革试点市和医养结合试点市建设深入推进，升级改造公办养老机构12家。成功举办国际滑雪邀请赛、“龙崛”国际格斗系列赛和辽阳首届文化体育产业博览交易会。深入实施“老字号”培育工程，汤河鲜鱼风味一条街晋升省级特色商业街区。加快发展“夜经济”和会展经济，举办各类促销活动150余次。

（四）加快国家现代农业示范区建设，农业农村发展基础进一步夯实

大力发展现代农业。全力推进9个现代农业示范区（带）建设，实施现代农业重点项目58个、乡镇（街）农业精品项目102个。调减玉米种植面积7.2万亩，粮食产量实现106万吨，耕种收综合机械化率83%。基本完成农村土地承包经营权确权登记颁证“整市推进”试点任务，新流转土地5.3万亩。大力发展适度规模经营，新建千亩以上土地股份合作社11家。加快发展农产品加工业，新增省级农事龙头企业6家。动物无害化处理中心投入使用，连续15年无区域性重大动物疫情发生。

持续完善农业农村基础设施。建设高标准农田11.1万亩，完成灯塔市万亩水稻省级试点任务。加速改造灯塔、浑沙灌区，实施农田水利维修工程172处。大中修、改扩建各级公路433公里，建设村内道路779公里。实施安全饮水工程89处，惠及19万农村人口。

全面启动特色小镇建设。皮草小镇晋升国家级特色小镇，温泉小镇、辽峰小镇等特色小镇建设成效显著。筹措资金8.2亿元，建设“升级版”美丽乡村示范村105个。

（五）全面深化改革扩大开放，体制机制更加完善

优化营商环境，推进“放管服”改革。市县两级全部成立行政审批局和营商环境建设监督局。大力实施简政放权，取消、调整、下放行政职权196项，通过再造流程压缩审批时限78%。“双随机、一公开”监管实现全覆盖。建立“智慧审批”模式，推行窗口单位延时服务，进一步便企利民。制定《优化营商环境条例》，落实“十严禁”纪律规定，深入开展七个专项行动，大力整治“七病一症”，亲商清商、守信践诺的营商环境初步形成。

深化重点领域改革。国有股全部退出工业竞争性领域，5家僵尸企业得到稳妥处置，“三供一业”分离移交任务基本完成，省下达公司制改革任务全面完成。国地税联合办税模式实现全覆盖。实行“多证合一”等商事制度改革，新增市场主体2.1万户。启动16家城市公立医院综合改革。化解政府直接债务34亿元。完成生产经营类事业单位改革。机关事业单位养老保险改革全面启动。推进城管执法体制改革，完成机构综合设置和执法重心下移。全面落实河长制，建立市县乡村四级河长体系。供销社、盐业等领域改革稳步推进。

提升对外开放水平。多措并举帮扶出口骨干企业，机电设备、服装等产品出口大幅增长。充分利用佟二堡保税仓库促进生皮进口。积极融入沈阳经济区，制定三年攻坚计划。主动开展与江苏南通对口合作，推动实施一批重点项目。

（六）加强城市建设和管理，生态文明建设再上新台阶

统筹推进新老城区建设。拆除东辽阳货站，启动宏伟路建设，扫清新老城区融合发展的最后屏障。万达广场、国际会展中心等商业设施投入运营，新城商业氛围日益浓厚。老城区改建力度不断加大，实施棚改项目38个，惠及居民1.1万户。综合改造老旧小区26个，修缮居民小区427个。实施绿化、美化、亮化工程，老城区整体形象明显提升。

加快城市基础设施建设。北环竣工通车，北部路网基本建成，西环和西部路网全面开工建设。打通2条断头路，完成新运大街电网落地工程，维修改造37条城市干道和街巷道路。河东交通综合枢纽一期如期竣工。实施南环街综合整治，建成南环景观游园。水电气热和环卫等基础设施建设不断加强。

全面强化城市管理。城市总规获国务院批准，控详规编制稳步推进。城市数字化管理平台初步建成，网格化、精细化管理不断完善。大力开展创城“八项整治” “十大行动”，集中整治马路市场、露天烧烤等违规行为，市容市貌不断改善。

加大环境保护力度。深入开展大气污染治理“十个专项行动”，实施最严格的秸秆禁烧制度，淘汰燃煤锅炉240台、黄标车及老旧车辆5000余辆，空气一级优天数增加30%。太子河生态治理初见成效。开展生态保护红线划定工作，国土空间开发格局不断优化。完成中央环保督察整改的序时任务。

（七）持续改善民生福祉，改革发展成果惠及广大群众

全面完成20项重点民生实事，市民幸福指数进一步提升。

大力开展脱贫攻坚。实施六大精准扶贫工程，稳定脱贫1万人，摘帽20个贫困村。

加强就业再就业工作。新增实名制就业1.6万人，城镇登记失业率3.5 %，零就业家庭保持动态为零。实施“高校毕业生就业促进计划”，应届毕业生就业率达到91%。

织密扎牢社会保障网。全面实施智慧人社工程，“惠民一卡通”手机APP功能全国领先。新农合人均保障标准提高到600元。城市低保、农村低保和农村特困人员供养标准分别提高6%、9%和7%。发放廉租房补贴3852户，改造农村危房1856户。千方百计筹措资金，确保养老金及时足额发放。

全面繁荣社会事业。各级各类教育均衡发展，一本上线率提高3%。启动健康辽阳建设，实施市县乡村健康一体化管理工程，医疗卫生服务水平不断提升。文化惠民活动丰富多彩，创排历史评剧《丁令威》，举办第六届全民读书节。培育4个省级历史文化街区，创建国家历史文化名城取得积极进展。国防动员、人防和民兵预备役建设进一步加强。统计、气象、防震减灾、档案、计生、老龄、慈善、红十字、妇女儿童、残疾人和地方志等事业取得新进步。

强化社会管理综合治理。全面建设立体化信息化社会治安防控体系，圆满完成党的十九大期间维稳安保任务。整合市民诉求平台，升级市长热线，为百姓提供更加便捷的服务。建立健全多元化群众诉求渠道，依法分类解决信访问题。安全生产总体形势持续保持平稳，群众饮食用药安全得到有效保障，产品质量安全水平进一步提升。国家安全、保密、民族宗教、外事侨务、涉台事务、应急管理等工作取得新成效，社会大局保持稳定。

（八）强化政府自身建设，执政能力不断提升

主动接受市人大及其常委会的法律监督、工作监督，认真执行市人大及其常委会的决议和决定；坚持重大事项决策前向人大报告，积极回应人大代表对社会热点问题的关注，办理人大代表建议115件。自觉接受市政协的民主监督，积极与政协开展民主协商，办理政协提案和建议案208件。认真听取各民主党派、工商联、无党派人士、人民团体的意见和建议，积极支持工会、共青团、妇联等人民团体依照各自章程开展工作。

深入学习贯彻习近平总书记系列重要讲话和党的十九大精神，积极开展“五包五促”活动，党员干部宗旨意识不断增强。开展“电视问政”11次，政风行风持续改善。加强法治政府建设，4部法规草案通过市人大常委会审议。“两学一做”学习教育步入常态化制度化。中央巡视“回头看”和省委巡视反馈问题得到有效解决。行政监察和审计监督不断强化。全面从严治党责任层层落实，风清气正的从政环境正在形成。

各位代表！回顾过去五年，成绩来之不易，是省委、省政府和市委正确领导、科学决策的结果，是全市人民团结奋斗、开拓进取的结果；是市人大法律监督、工作监督和市政协民主监督、参政议政的结果；是驻军部队，中央、省驻我市单位，各民主党派、人民团体、社会各界人士、外来投资者和建设者关心支持的结果。在此，我代表市政府，向参与辽阳经济社会建设的全市人民和各界人士表示衷心感谢！

各位代表！成绩值得肯定，问题不容忽视。一是传统产业转型升级步伐不快，新动能支撑力尚未形成，骨干企业不多，产业链条还不完善。二是体制机制障碍没有得到根本解决，重点领域和关键环节改革仍需突破。三是引领创新发展能力不强，以企业为主体的创新体系还不健全。四是教育、医疗、住房、环保等领域的民生问题还较多，保障和改善民生任重道远。五是政府自身建设还需进一步加强。对照新常态、新要求，在发展理念、思想观念上还有差距，在服务意识和工作作风上仍有欠缺，不敢担当、不愿作为的现象时有发生，政府重大风险防控能力有待加强。对此，我们一定要坚持问题导向，采取有力措施，认真加以解决。

二、今后五年工作总体安排和2018年目标任务

今后五年是我市全面落实党的十九大精神、决胜全面建成小康社会、加速“四个辽阳”建设的关键时期。我们要在省委、省政府和市委的正确领导下，抢抓机遇，乘势而上，以更大的勇气、更实的作风，全力推动新一轮振兴发展。

今后五年政府工作的指导思想是：高举习近平新时代中国特色社会主义思想伟大旗帜，全面贯彻党的十九大精神，坚持稳中求进工作总基调，按照高质量发展的要求，统筹推进“五位一体”总体布局，协调推进“四个全面”战略布局，持之以恒落实新发展理念和“四个着力”“三个推进”，加快实施市十二次党代会各项部署，把握引领经济发展新常态，加快建设现代化经济体系，高标准融入沈阳经济区，大力实施乡村振兴战略，加速城乡一体化进程，全力保障和改善民生，加强生态文明建设，走出一条质量更高、效益更好、结构更优、环境更佳、活力更强的发展新路，确保全面建成小康社会，实现“十三五”规划目标，大幅提升“四个辽阳”建设水平，开启全面建设社会主义现代化新征程，谱写振兴发展新篇章。

今后五年的总体目标是：体制机制进一步完善，重点领域改革取得实效，沈阳经济区中心城市的“后花园”和“卫星城”初步建成；供给侧结构性改革深入推进，经济结构进一步优化，实体经济健康平稳发展，现代化经济体系加速建立；创新型城市全面建成，大众创业、万众创新的氛围日益浓厚；生态环境质量总体改善，城镇化水平显著提高，人民生活更加富庶，社会更加文明和谐稳定。通过努力，全面建成小康社会，“四个辽阳”建设取得重大成果，新一轮振兴发展迈出新步伐。

今后五年的主要任务是：

——着力完善体制机制，增强经济发展动力。加快形成同市场完全对接、充满内在活力的新体制和新机制。持续优化营商环境，努力构建“亲”“清”新型政商关系。加快转变政府职能，大力推进“放管服”改革。全面深化国资国企、政府机构和行政体制等重点领域改革。主动对接“一带一路”战略，打造对外竞争新优势。加快沈阳经济区建设，实施一体化发展共同行动计划，共同建设“五个中心”，共同打造新型工业化示范区及具有较强竞争力和影响力的城市群。

——着力推进结构调整，增强经济质量优势。深入实施《中国制造2025辽阳行动纲要》，推动互联网、大数据、人工智能与实体经济深度融合，支持重点产业、龙头企业做大做强，建成“两大千亿元产业基地、一大产业集群”，促进新旧动能接续转换。加快发展现代服务业，全面提升6个省级服务业集聚区综合实力，发展壮大“冰雪温泉、皮草购物、历史文化、自然生态”四大特色产业。深入实施乡村振兴战略，推动农业全面升级、农村全面进步、农民全面发展。高标准建成国家现代农业示范区，基本实现农业现代化。加快县域经济发展，力争灯塔市进入全国百强县，辽阳县全省排名“晋位升级”。

——着力鼓励创新创业，增强创新发展能力。加快构建以企业为主体、市场为导向、产学研深度融合的技术创新体系，推动形成区域性创新中心。强化产业技术体系创新，运用新技术新业态改造提升传统产业。促进技术创新和管理创新、商业模式创新融合，发展数字经济、智能经济。大力推动创新创业，支持创新型企业发展。积极融入沈大国家自主创新示范区，全面提升高新区创新能力。大力培养和引进高端人才，加强企业家和工匠队伍建设，营造“创新无禁区、创业无障碍”的发展环境。到2022年，高新技术企业达到150家以上，建设各级各类创新平台、研发机构150家以上，研发经费投入占GDP比重达到2.2%。

——着力保障和改善民生，增强群众获得感、幸福感、安全感。坚持在经济增长的同时实现居民收入同步增长。坚决打赢脱贫攻坚战。实施更加积极的就业政策，不断完善社会保障体系。运用“互联网+”，全面提升教育、卫生、文化、体育等公共服务均等化、普惠化、便捷化水平。实行最严格的生态环境保护制度，坚决打赢大气、水、土壤污染防治攻坚战。推动新老城区融合发展，加快城乡一体化进程，常住人口城镇化率达到66%。社会治理制度不断健全，法治建设、平安建设全面推进。建成国家历史文化名城、国家森林城市和国家卫生城市，继续争创全国文明城市和全国双拥模范城。

各位代表！未来五年发展蓝图已经绘就，目标任务已经明确。我们要大力推进作风转变，以锲而不舍的钉钉子精神，一件事情接着一件事情办、一年接着一年干，持之以恒、久久为功。我们要不断增强学习本领、政治领导本领、改革创新本领、科学发展本领、依法行政本领、群众工作本领、狠抓落实本领、驾驭风险本领，落实全面从严治党主体责任，切实加强政府自身建设，努力打造人民满意的服务型政府，为新一轮振兴发展提供有力保障。

各位代表！2018年是全面贯彻党的十九大精神的开局之年，是改革开放40周年，是决胜全面建成小康社会、实施“十三五”规划承上启下、推动“四个辽阳”建设迈上新台阶的关键之年。我们要认真贯彻落实中央、省委、市委经济工作会议精神，坚持稳中求进工作总基调，坚定信心，汇聚力量，奋力拼搏，开创振兴发展新局面。

今年经济社会发展的主要预期目标是：地区生产总值增长6.5%左右，一般公共预算收入增长7%左右，固定资产投资增长10%左右，城乡常住居民人均可支配收入均增长6.5%左右。

我们要突出抓好以下九方面工作：

（一）切实发挥投资和消费的有效作用，促进经济发展持续向好，强化“四个辽阳”建设支撑

发挥投资对优化供给结构的关键性作用。继续开展“项目年”活动，实施投资亿元以上项目146个。围绕“两大千亿元产业基地、一大产业集群”建设，再谋划推进一批重点项目。加强园区基础设施建设，提高承载能力。放宽市场准入条件，规范政府和社会资本合作，促进民间投资合理增长。大力争取上级资金，全年到位资金90亿元以上。推动“银政企保”合作，建立融资担保体系，融资担保能力达到100亿元以上。实施“双招双引”行动计划，精准引资引才政策，推动一批项目签约落地，吸引一批高端人才落户。

增强消费对经济发展的基础作用。增加高品质产品和服务的有效供给，提升居民消费水平。稳步推进住房消费，加快棚户区改造，全面实行货币化安置，健全完善租购并举的住房制度，有计划供应土地，引导房地产市场健康有序发展。搞活辽阳大剧院、健身大厦等文体场馆，市场化运作高水平赛事、大型演出等活动，提升文体消费水平。加快全国养老服务业综合改革和医养结合两个试点市建设，发展壮大民办养老机构，推进居家社区养老服务全覆盖。加快发展连锁经营和特许加盟，积极培育“老字号”。大力发展“夜经济”“假日经济”和会展经济，再创建一批省级特色街区。

（二）深入推进供给侧结构性改革，进一步增强经济发展质量和效益，激活“四个辽阳”建设动能

大力振兴实体经济。完善民营经济发展优惠政策，全面推进民营经济发展改革示范试点六项工作。落实减税降费政策，进一步减轻企业负担。统筹运用“过桥”资金、助保贷、产业投资基金等手段，加大金融对实体经济的支持力度。推动企业上市，扩大直接融资规模。激发和保护企业家精神，弘扬劳模和工匠精神，实施具有国际视野的创新型企业家培养工程，造就优秀企业家队伍。坚持定期联系企业家制度，更好支持企业家创新创业。深入实施“个转企”“小升规”“大做强”工程，壮大实体经济规模。

加快工业转型升级步伐。深入实施《中国制造2025辽阳行动纲要》，推动产业发展迈向中高端。做精做优芳烃及精细化工产业基地，确保辽化大技改等项目如期投产达效，实施高性能聚丙烯等22个项目建设，拉长加粗产业链条。做大做强铝合金精深加工产业基地，确保专用车厢项目全部达产，加快推进200万吨铝板带箔等6个项目建设，引进一批关联企业，提升产业丰厚度。发展壮大装备制造及汽车零部件产业集群，实施年产6万台充电桩等22个项目，加快中欧产业园和“双创”产业园建设。实施消费品工业“三品”工程，支持钢铁企业改造升级，推动水泥企业兼并重组。大力发展智能制造，加速“两化”融合。

推动服务业提档升级。围绕6个省级服务业集聚区，积极引进新的商业业态，进一步优化商业布局。树立“旅游+”理念，创新旅游宣传和服务手段，提升旅游知名度。充分发挥旅游集团作用，推进东京城遗址公园、核伙沟等景区建设。设置旅游标识牌，实施“旅游厕所革命”，加快房车营地建设，完善旅游服务功能。制定实施民宿经济发展意见，鼓励和规范“农家乐”发展。大力发展金融业，加快白塔金融集聚区建设，推动符合条件的金融机构依规开展新业务，再引进一批金融机构。加快现代综合物流园区建设，开展跨境冷链班列业务。完成农产品现代流通体系和肉菜流通追溯体系两个试点市建设任务。支持电商企业强强联合，网络交易额实现30亿元。

（三）实施乡村振兴战略，加快推进农业农村现代化，夯实“四个辽阳”建设基础

加快国家现代农业示范区建设。巩固9个现代农业示范区（带）建设成果，启动太子河休闲农业示范带建设，实施一批农业精品项目。大力发展都市观光农业、养生休闲农业，打造城郊型现代农业示范区。

加快农村一二三产业融合发展。主动对接市场需求，调优农产品品种，提升农产品质量。调整种植业结构，调减玉米种植面积3万亩以上，新发展设施农业1万亩以上。稳定粮食产量，确保粮食安全。支持养殖业发展，培育壮大一批龙头企业。深化农村改革，加快土地流转。积极培育家庭农场、种养大户、农民专业合作社、农业企业等新型经营主体，采取多种经营方式，提高适度规模经营水平。大力发展农业产业化，新建一批重点项目，推进佟二堡省级农产品加工集聚区建设。积极发展农村电子商务和物流业，着力打通“工业品下乡”“农产品进城”双向流通渠道。加大金融支持“三农”力度，努力增加农业农村贷款。加强动植物疫病防控，确保不发生区域性重大疫情。

进一步完善农业农村基础设施。新建高标准农田9.5万亩，加快灌区改造步伐，实施水库移民工程59项。大中修、改扩建各级公路248公里。再解决一批农村安全饮水问题。实施农村人居环境整治行动，建设美丽宜居乡村。

大力发展县域经济。实施县域经济发展三年行动计划，壮大县域经济总量。改革和完善县乡财政体制，调动乡镇发展经济、培植财源、加强征管的积极性。破解资金瓶颈问题，加快特色小镇建设。

（四）深化重点领域改革，推动形成全面开放格局，增强“四个辽阳”建设动力

全面优化营商环境。进一步简政放权，完善权责清单。深化行政审批制度改革，持续完善和再造审批流程。提升“双随机、一公开”监管水平，推动形成以信用为核心的监管新模式。加快推进“互联网+政务服务”，实现全市行政审批“一张网”。优化政府采购、工程招标交易流程，建成全市公共资源交易平台。继续开展营商环境建设年活动，深入整治“七病一症”，力争实现市场主体、税收、投诉“两增一减”，营造更好、更优的“辽阳气候”。

深化重点领域改革。因企施策，依法处置僵尸企业。稳步实施厂办大集体改革，做好“三供一业”分离移交后续工作，稳妥推进国有企业混合所有制改革。加强国有资产监管，防止资产流失。深入推进市与县（市）区财政事权和支出责任划分改革，适时启动消费税体制改革。防范地方金融风险，化解存量政府债务。加快实施基本公共卫生服务和基本药物制度。深化公立医院综合改革，逐步建立现代医院管理制度。加强信用体系建设，加快构建守信联合激励和失信联合惩戒机制。全面推行河长制，出台实施《河长制实施方案》和《一河一策治理及保护方案》。创新产业园区体制机制。继续推进商事、事业单位、供销社等领域改革。

全面提升对外开放水平。加快出口基地建设，壮大出口规模。提升出口质量和附加值，促进更多优质产品打入国际市场。积极融入“一带一路”战略，支持企业开展境外投资。落实一批利用外资项目，带动企业嵌入全球链、价值链、创新链。全面实施沈阳经济区建设发展辽阳三年攻坚计划，推进与沈阳产业对接、要素整合、政策共享。按照现代化国际化城市的标准，推进与沈阳同质化同城化发展。加强与江苏南通对口合作。

（五）加强城市规划建设管理，加速新型城镇化进程，展现“四个辽阳”建设风貌

坚持规划引领。启动新一轮城市总规修编，高标准编制重点区域城市设计和控详规，进一步完善各类专项规划，划定生态控制线和城镇开发边界。加快推进“多规合一”，切实增强城市发展的整体性、系统性和生长性。

不断完善城市基础设施。加速东辽阳老工业区搬迁改造，全线贯通宏伟路。加快推进大型人防公交枢纽、西环、西部路网等基础设施建设，确保北部路网竣工通车。维修改造一批街巷道路，继续打通城市断头路。扩容改造垃圾处理场，启动垃圾焚烧发电项目前期工作。继续实施绿化、美化、亮化工程，加快燃气、供水、供电、供热等基础设施建设。

全面加强城市治理。巩固全国文明城市创建成果，持续开展城管执法专项整治行动，大力推进创建工作常态化。深化城管执法体制改革，完成新增执法职权划转移交任务，全部下放适合区政府行使的城市建设管理权限。

加速城乡一体化进程。加快农业转移人口市民化，促进农民工在城镇落户，享受公共服务均等化待遇。完善乡镇规划，严格规划实施，坚持因地制宜、一镇一策，加速推进佟二堡、小北河等省级特色乡镇建设，培育建设一批宜业、宜居、宜游乡镇。

（六）深入实施创新驱动战略，打造竞争新优势，培育“四个辽阳”建设引擎

促进科技成果转化。加强与“两院十校”对接，推进产学研合作和协同创新，着力打造沈阳科技成果转化的承接地。加快国家、省重点实验室和中试基地建设，推动钢铁共性技术创新中心与企业良性互动。完善科技成果转化O2O服务平台，促成科技成果转化项目30项。

打造创新高地。完善鼓励科技创新意见，营造浓厚的创新氛围。做大做强科技信贷担保资金规模，推动企业增加科技投入。支持向阳工业集中区晋升省级高新区、辽峰小镇争创省级农高区。新增高新技术企业20家。

推动大众创业、万众创新。发挥高端人才队伍创新引领作用，争取更多高端人才落户我市。选派30名工业科技特派员和专家入驻重点企业，提高企业创新能力。建设一批省市级双创示范基地。落实创业担保贷款、税费减免、创业培训补贴等政策，助力市场主体创业发展。

（七）加大环境保护和治理力度，切实优化生态环境，坚守 “四个辽阳”建设理念

全面治理大气污染。持续开展“清风治霾”行动，打好蓝天保卫战。推广清洁能源，淘汰燃煤锅炉111台，实施农村“煤改气”试点工程。全面加强秸秆禁烧工作，大力推进秸秆综合利用。

加强水污染防治。因河施策，实施污染治理，确保6个国控河流断面全部达标，省控以下河流断面水质明显改善，完成“水十条”年度工作任务。深入实施太子河生态治理工程。

保护土壤环境。完成农用地土壤污染详查，启动工业用地土壤污染详查。建立健全土壤环境质量监测网络，有效控制和消除土壤污染源，推动土壤环境质量稳中向好。

创建国家森林城市。实施城市绿地系统、道路绿量、村屯绿化等重点工程，荒山造林5600亩，封山育林2万亩，建成森林城市科普馆。加快建设太子河、柳壕河湿地公园。实施双河及周边地区生态修复建设总体规划，启动一期工程。

完成生态保护红线划定工作，编制自然资源资产负债表，开展领导干部自然资源资产离任审计，筑牢绿水青山就是金山银山的理念。

（八）提高保障和改善民生水平，加强和创新社会治理，共享“四个辽阳”建设成果

打好脱贫攻坚战。巩固已有脱贫成果，再精准脱贫1860人、销号12个贫困村。

提升就业质量，加强社会保障体系建设。新增城镇实名制就业1.5万人，城镇登记失业率控制在3.5%以内。实施城乡居民和各类群体收入增长三年行动计划。完善机关事业单位养老保险制度。城乡低保标准均增长5%以上。加快基本医疗保险改革，巩固大病保险制度，推进异地就医联网结算。深入实施养老服务惠民工程，让广大老年人受益。促进慈善、红十字等事业健康发展。防范养老金风险，落实提标政策，确保及时足额发放。

优先发展教育事业。促进义务教育均衡发展，推动辽阳县、灯塔市创建国家义务教育均衡县。继续深化协作化办学，有效发挥“三通两平台”作用，让广大师生充分享受优质教育资源。加大薄弱学校改造力度。积极引进社会资本建设普惠制幼儿园。加强教师队伍和校长队伍建设。

深入实施健康辽阳建设。树立大健康理念，开展“十大健康行动”，积极创建国家卫生城市。强化重大疾病防控，加快基层医疗机构建设，进一步提高城乡居民医保补助标准，增强基本公共卫生服务能力。

积极发展文化体育事业。完善公共文化服务体系，大力推进文化下乡，强化基层场所建设。提升竞技体育综合实力，广泛开展群众性文化体育活动。加快创建国家历史文化名城。

打造共建共治共享的社会治理格局。完善城乡社会治安防控体系，努力建设更高水平的平安辽阳。集中化解信访积案，减少社会不稳定因素。强化生产、消防、交通、产品质量、食品药品等安全监管，提高突发事件应急处置和防灾减灾能力。深入开展“放心消费”创建活动，切实保障消费者权益。加强全民国防教育和国防动员建设。创新社区管理，实施社区工作者素质提升工程。

（九）增强执政本领，努力建设人民满意的服务型政府，凝聚“四个辽阳”建设合力

加强政治建设。坚定理想信念，树牢“四个意识”，增强“四个自信”，坚决维护以习近平同志为核心的党中央权威和集中统一领导。不折不扣贯彻落实上级各项部署要求。开展“不忘初心、牢记使命”专题教育，推进“两学一做”学习教育制度化常态化。

加强法治建设。把法治理念落实到政府工作全领域、全过程，努力打造法治政府。自觉接受市人大及其常委会法律监督、工作监督和市政协民主监督。认真办理好人大代表议案、建议和政协委员提案。充分听取各民主党派、工商联、无党派人士和工会、共青团、妇联等人民团体的意见建议。

加强效能建设。充分利用协同办公系统，进一步提高办公效率。开展履职培训，增强服务能力。加强督查力量，完善督查制度，提高督查水平。继续实施绩效考核，落实奖惩政策，激发工作动力。

加强作风建设。坚持说实话、谋实事、出实招、求实效。时刻警惕形式主义、官僚主义十种新的表现形式，坚决防止“四风”问题回潮。大兴调查研究之风，求真务实、真抓实干。深入开展“五包五促”活动，进一步密切党群干群关系。

加强廉政建设。认真贯彻落实中央八项规定精神及省市《实施细则》。加强审计监督，促进廉洁依法行政。落实全面从严治党主体责任，加强党风廉政建设，树立清正廉洁的良好形象。

各位代表！人民对美好生活的向往就是我们的奋斗目标。今年，市政府要在全面做好各项民生工作的基础上，着力办好20项重点民生实事，让群众获得感、幸福感、安全感更加充实。

各位代表！新思想引领新时代，新时代开启新征程。让我们紧密团结在以习近平同志为核心的党中央周围，在省委、省政府和市委的正确领导下，抢抓机遇，攻坚克难，全面建设智慧文明美丽幸福新辽阳，为新一轮振兴发展作出新的更大的贡献！
